# Prehabilitation for men undergoing radical prostatectomy: a multi-centre, pilot randomized controlled trial

**DOI:** 10.1186/1471-2482-14-89

**Published:** 2014-11-13

**Authors:** Daniel Santa Mina, Andrew G Matthew, William J Hilton, Darren Au, Rashami Awasthi, Shabbir MH Alibhai, Hance Clarke, Paul Ritvo, John Trachtenberg, Neil E Fleshner, Antonio Finelli, Duminda Wijeysundera, Armen Aprikian, Simon Tanguay, Franco Carli

**Affiliations:** Kinesiology Program, University of Guelph-Humber, Toronto, ON Canada; Prostate Centre, Princess Margaret Cancer Centre, Toronto, ON Canada; University of Guelph, Guelph, ON Canada; University of Toronto, Toronto, ON Canada; McGill University Health Centre, Montreal, QC Canada; University Health Network, Toronto, ON Canada; Kinesiology and Health Sciences Department, York University, Toronto, ON Canada; Cancer Care Ontario, Toronto, ON Canada

**Keywords:** Prehabilitation, Prostate cancer, Exercise, Randomized controlled trial, Rehabilitation

## Abstract

**Background:**

An emerging field of research describes the role of *pre*operative health behaviours, known as prehabilitation. The preoperative period may be a more physically and emotionally salient time to introduce and foster chronic adherence to health behaviours, such as exercise, in patients compared to post-treatment during recovery. Moreover, physical and psychosocial improvements during the preoperative period may translate into an enhanced recovery trajectory with reduced operative complications and postoperative adverse effects. No studies have assessed prehabilitation for men with prostate cancer undergoing radical prostatectomy.

**Methods/Design:**

This is a multi-centre, pilot randomized control trial conducted at two Canadian urban teaching hospitals. 100 men undergoing radical prostatectomy for prostate cancer with no contraindications to exercise will be recruited and randomized to the prehabiliation program or usual care. Prehabilitation participants will engage in a preoperative, individualized exercise program including pelvic floor muscle strengthening instructions and a healthy lifestyle guide for men with prostate cancer. These participants will be asked to engage in 60 minutes of home-based, unsupervised, moderate-intensity exercise on 3–4 days per week. Usual care participants will receive the same pelvic floor muscle strengthening instructions and healthy lifestyle guide only. We will assess the feasibility of conducting an adequately powered trial of the same design via recruitment rate, programmatic adherence/contamination, attrition, and safety. Estimates of intervention efficacy will be captured through measurements at baseline (4–8 weeks preoperatively), within 1 week prior to surgery, and postoperatively at 4, 12, and 26 weeks. Efficacy outcomes include: fatigue, quality of life, urinary incontinence, physical fitness, body composition, aerobic fitness, pain, and physical activity volume.

**Discussion:**

The primary outcome of this study is to determine the feasibility of conducting a full-scale, randomized controlled trial of prehabilitation versus usual care and to estimate effect sizes that will inform sample size determinations for subsequent trials in this field. To our knowledge, this is the first study to examine a structured presurgical exercise program for men undergoing radical prostatectomy for prostate cancer. This trial will advance our understanding of strategies to efficiently and effectively use the preoperative period to optimize postoperative recovery.

**Trial registration:**

Clinicaltrials.gov Identifier: NCT02036684

## Background

Radical prostatectomy (RP) is the most common and effective treatment for localized prostate cancer (PCa), with a 15-year survival rate of approximately 90% [1, 2]. Unfortunately, RP is associated with significant adverse effects, such as urinary incontinence, sexual dysfunction, and reduced physical function that collectively diminish health-related quality of life (HRQOL); such decrements may persist for up to two years postoperatively [3, 4]. Given the efficacy of RP and its widespread use in the management of PCa, it is suggested that the metric of surgical success should include more than simply disease-free survival; rather, it should include a comprehensive assessment of physical and psychosocial wellbeing and the rate at which the patient returns to baseline levels of wellbeing [1, 5].

Traditionally, interventions intended to improve RP recovery and minimize adverse effects were limited to the postoperative period. Such approaches have typically focused on the urological side effects of urinary incontinence and sexual dysfunction through pelvic floor muscle exercises (PFMX) and/or phosphodiesterase-5 inhibitors while psychotherapy is commonly employed for psychological adjustments in the postoperative period [5–8]. Overall, research suggests that patients are often non-adherent to the prescribed postoperative interventions [9]. Furthermore, little attention has been directed towards the general physical and functional declines that RP patients experience, despite their significant contribution to overall HRQOL [10, 11]. Strassels et al. [12] found that physical functioning one month post-RP was below population norms and that 43% were dissatisfied with their physical ability. At 3 months post-RP, Litwin et al. [13] found that only 30% and 36% of patients had returned to baseline values of physical function and energy levels, respectively. Given that the recovery from RP may be blunted by postoperative decreases in physical functioning and concomitant non-compliance with traditional post-RP treatments, examination of interventions applied in the pre-surgical phase is warranted.

To enhance the overall surgical experience for patients, an important question revolves around *when* the most opportune time is to introduce recovery-optimizing behaviours. The postoperative period may be less than ideal due to concerns related to perturbing the healing process as patients await additional results or treatments. Instead, an emerging field of research describes the role of preoperative strategies to improve treatment tolerance and recovery. The preoperative period may be more physically and emotionally salient for patients by capitalizing on: i) the generally better physical condition of the patient (compared to the acute postoperative period), ii) surgical wait-list times, and iii) a ‘teachable moment’ for the patient that accompanies the need for major surgery [14]. Ultimately, it appears that the preoperative period may be an optimal time to invest into the modifiable factors that contribute to peri- and postoperative health.

A health behaviour that has demonstrated great promise in terms of its beneficial relationship with perioperative complications and postoperative recovery is physical activity and the consequential improved physical fitness levels. Studies routinely report that patients who are physically active and fit recover more quickly, have fewer perioperative complications, and experience better convalescence compared with patients who are less physically active and fit [15–21]. *Pre*-habilitation is defined as the process of enhancing one’s functional and mental capacity to enable him/her to withstand a significant stressor [22]. In a surgical setting, preoperative physical and/or psychological conditioning aims to increase body and mind reserves to prevent the declines in postoperative wellbeing [22]. Recently published systematic reviews of prehabilitation have described numerous benefits to postoperative wellbeing across a variety of surgical populations [23–25], including cancer-patients specifically [26]. Carli and colleagues have shown that prehabilitation can improve postoperative physical function in cancer patients undergoing colorectal surgery [27, 28], hysterectomy [29], and lung resection [30]. Their group has also found that improvements in physical function were associated with improvements in mental health, vitality, and self-perceived health [31]. Moreover, it appears that control subjects whose fitness deteriorated preoperatively had more surgical complications and greater need for intensive care [31].

With respect to PCa patients undergoing RP, we have previously shown in an observational study that physical activity in the year prior to RP is associated with improved postoperative HRQOL [32]. Additionally, in a cohort of 509 patients undergoing RP, we observed that men who were meeting physical activity guidelines prior to surgery had greater HRQOL at 6 and 26 weeks postoperatively compared to men who were not meeting the PA guidelines [33]. Moreover, we found that men who were active preoperatively were less likely to report being incontinent 6 weeks after surgery than their inactive counterparts. Exercises targeted at the pelvic floor have also shown benefit for RP patients as two studies have demonstrated effectiveness at improving urinary incontinence by 20% compared to controls [34, 35]. These studies have revealed an important role for physical activity and fitness in the postoperative wellbeing of men undergoing RP; however, no randomized controlled trial has been conducted to determine a causal relationship between preoperative exercise and postoperative outcomes in this population. Thus, there remains an important opportunity to examine a comprehensive prehabilitation program for men undergoing RP for PCa that may have a profound influence on surgical preparation by reducing the often-chronic nature of complete recovery from this procedure.

## Methods/Design

This study is a 2-arm, multi-centre, pilot randomized controlled trial to examine the effect of a comprehensive prehabilitation program (PREHAB) versus usual care (UC). The primary objective of this study is to assess the feasibility of conducting of an adequately powered study of similar design. Our secondary objective is to report estimates of efficacy on several clinically important outcomes for RP patients.

This study will be conducted at two urban Canadian teaching hospitals: the Princess Margaret Cancer Centre in Toronto, Ontario, and the McGill University Health Centre in Montreal, Quebec. Both participating institutions have received approval from their respective research ethics boards.

### Participants

We will recruit 100 participants (n = 50 per site and per group) consistent with recommended sample sizes for a pilot study [36]. We anticipate an attrition rate of 20%. *Inclusion criteria:* Men aged 40 and 80 years of age with localized PCa (stage cT1- cT2) who have consented for RP and are proficient in English or French. *Exclusion criteria*: i) severe coronary artery disease (Canadian Cardiovascular Society class III or greater); ii) significant congestive heart failure (New York Heart Association class III or greater); iii) uncontrolled pain; iv) neurological or musculoskeletal co-morbidity inhibiting exercise; v) diagnosed psychotic, addictive, or major cognitive disorders; vi) no more than two American College of Sports Medicine Coronary Risk Factors [37].

### Study recruitment and randomization

Eligible patients will be recruited from ambulatory urology clinics at each site by a research coordinator (RC). Recruitment posters will also be placed in hospital waiting areas. Written informed consent will be collected from all patients prior to participation.

**Figure 1:**
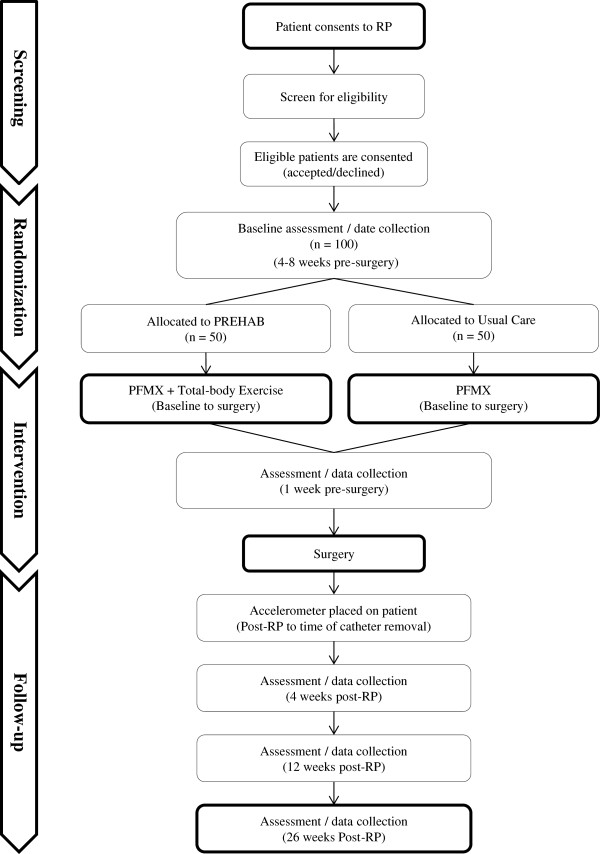
**Participant flow.**

Participants will be randomly allocated to either the PREHAB or UC groups prior to the baseline assessment, stratified by study site. Blinded allocation of participants to their treatment groups will be performed via sequentially numbered opaque envelopes that will contain intervention assignments that have been shuffled to create a random order. Figure 1 provides the structure of participant flow through each intervention arm.

### Study arms

Both groups will begin participation in their respective study arms at the time of randomization following shortly after RP scheduling. The duration of the preoperative wait-time (typically 4-8 weeks) will be recorded. Both groups will receive a copy of a PCa-specific lifestyle support book containing information on topics such as nutrition, active-living, and pelvic floor training [38].

#### PREHAB

PREHAB participants will engage in an individualized, total-body exercise and PFMX program. The total-body exercise prescription will consist of 60 minutes of home-based, unsupervised exercise on 3–4 days per week. Each session will be individualized, based upon a baseline assessment and will include: a 5-minute warm-up, 25 minutes of aerobic exercise (40-60% of heart rate reserve; HRR), 25 minutes of resistance training (5 exercises targeting major muscle groups performed at an intensity of 8–12 repetitions maximum), and a 5-minute cool-down. Training intensity progression will occur when the participant can complete aerobic exercise with mild exertion and/or when the participant can complete 15 repetitions of a given resistance exercise. Participants will be provided with varying intensities of resistance bands, a stability ball, and an exercise mat. The PFMX prescription will begin with instructions on how to engage the pelvic floor delivered by the RC trained in PFMX. The PFMX prescription will include a gradual increase in repetitions from 60 per day during weeks 1–2, 120 per day during weeks 3–4, and 180 per day during week 5 to the surgical date. The total number of repetitions of the PFMX will be divided equally between the rhythmic contractions (contract and relax over one second) and the sustained contractions (contract and hold for up to 10 seconds). Participants will be instructed to contract with maximal effort during all PFMX repetitions. The PREHAB intervention will be supported with a manual (translated into French) and exercise videos created by the co-investigating team. The RC will communicate with the PREHAB participants weekly via phone or email to ensure program compliance, support appropriate progression, and address any barriers to exercise that may prevent ongoing participation.

#### Usual care

RP patients will receive preoperative information from a urology nurse educator about PFMX, mobilization and general timeframes for return to normal activities following RP. The UC group will receive the same PFMX prescription as the PREHAB intervention and will receive weekly communication from the RC regarding compliance with the PFMX prescription to provide an attentional-control.

### Outcome assessments

Self-report measures and physical fitness assessments will be conducted at: baseline (following RP booking, prior to beginning group assignment) approximately 4–8 weeks preoperatively, within 1 week prior to RP, and at 4, 12, and 26 weeks postoperatively. All self-reported measures are available in English and French.

Full-scale trial feasibility will be assessed by: 1) recruitment rate (and reasons for non-participation); 2) attrition rate; and 3) adherence and contamination (through a physical activity log completed by the RC during the weekly communication). Adherence to the exercise prescription for the PREHAB intervention and to the PFMX prescription for both groups will be analyzed by calculating the percentage of total physical activity volume prescribed (i.e. total number of repetitions completed/total number of repetitions prescribed).

Musculoskeletal fitness will be assessed by grip strength dynamometry and isometric strength for elbow flexion and extension by handheld dynamometry. Grip strength will be measured using a hand grip dynamometer (Sammons Preston, model Jamar, Bolingbrook, IL, USA) with the participant asked to complete two maximal effort squeezes per hand while standing and arm extended and abducted to 45 degrees [39]. Grip strength will be recorded to the nearest kilogram, with the maximum value per hand used for outcome assessment. Isometric strength for elbow flexion and extension will be measured using a handheld digital dynamometer (Hoggan Health Industries, model Microfet2, UT, USA) that is positioned on the underside (for flexion) or topside (for extension) a table. Participants will be seated with elbow flexed to 90 degrees, with the dynamometer in hand, either on the under or topside of table and will gradually generate force against the dynamometer for 2 seconds, and then maintains a maximal effort for another 5 seconds. For elbow flexion, the dynamometer is positioned in the participant’s hand, with the participant pressing the device upwards, onto the underside of the table. For elbow extension, the dynamometer placed in the participant’s hand, with the participant pressing the device downwards, onto the topside of the table. For elbow extension, the dynamometer placed proximal to wrist on extensor surface of forearm, with the participant pressing downwards, into the dynamometer. The participant will complete two trials per arm with the maximum value per arm recorded to the nearest kilogram. Waist circumference (WC) will be measured according to the World Health Organization protocol, with measuring tape positioned at the midpoint between lowest rib and iliac crest Body mass index (BMI, kg/m^2^) will be calculated using the participant’s height (m) and weight (kg). Body fat percentage will be measured through bioelectrical impedance analysis (Tanita Corporation, model TBF-300A, Tokyo, Japan). Aerobic fitness will be measured using the 6-Minute Walk Test (6MWT) [40] that involves the participant walking a 15 meter linear course for 6 minutes (turning around at each end) with the total distance traveled recorded. Motivational and time-remaining cues are standardized to the following:

1:00 – “You’re doing well. You have 5 minutes to go”.

2:00 – “Keep up the good work. You have 4 minutes to go”.

3:00 – “You’re doing well. You’re halfway done”.

4:00 – “Keep you the good work. You have 2 minutes left”.

5:00 – “You’re doing well. You have only 1 minute to go”.

5:45 – “In a moment I’m going to tell you to stop. When I do, just stop right where you are and I will come to you”.

PCa-specific HRQOL will be measured using the psychometrically validated Functional Assessment of Cancer Therapy-Prostate (FACT-P) [41] and the Patient-Oriented Prostate Utility Scale (PORPUS) [42]. Anxiety and depression will be measured by the Hospital Anxiety and Depression Scale (HADS) [43]. The HADS demonstrates strong construct validity and is sensitive to a counseling intervention [44, 45]. Cancer-specific fatigue will be measured using the Functional Assessment of Cancer Therapy-Fatigue (FACT–F) which is a widely used 13-item measure with strong reliability and validity [45, 46]. The Pain Disability Index (PDI) [47] will be used to assess the extent to which persistent pain interferes with an individual’s ability to engage in activities of daily living [48]. Urological symptoms are assessed using the valid and reliable, 7-item International Prostate Symptom Score (IPSS) [49, 50]. Erectile function is assessed using the 5-item International Index of Erectile Function (IIEF) scale, a widely used, psychometrically validated multi-dimensional self-report instrument evaluating male sexual function [51, 52].

Physical activity level will be measured at each time point through the Community Health Activities Model Program for Seniors (CHAMPS) questionnaire [53]. The CHAMPS is a self-reported measure of physical activity, comprising 41 activities evaluated according to the total number of hours done during an average week that can be translated into average weekly caloric expenditure. Acute postoperative physical activity will also be objectively measured via accelerometer from postoperative admission to the inpatient ward until the participant returns to the ambulatory clinic for catheter removal (typically 1–2 weeks). The Actiwatch 2 (Philips Healthcare, Respironics, PA, USA) is a light-weight accelerometer worn as a wristwatch on the non-dominant arm that is water resistant and can be worn during bathing. The Actiwatch 2 can reliably measure movement volume in three dimensions and quantified using the unit of ‘activity counts’ (AC) over multiple days [54–56]. ACs (in counts per minute; cpm) will be captured and stratified into the following categories for analysis: sedentary (0–99 cpm), light activity (100–1951 cpm), and moderate-to-vigorous activity (>1951 cpm). As reported by Lynch et al. [57], physical activity volume will be analyzed using percentage of device-wearing time (in minutes) spent in the various activity categories.

Treatment complications and length of stay data will be extracted from medical records.

### Statistical analysis

Participant characteristics will be summarized using descriptive statistics (mean, standard deviation, frequency). The equivalence of groups at baseline in terms of demographic and clinical variables will be assessed using independent samples t-tests for continuous variables and chi-square tests for categorical variables. We will report recruitment and attrition rates per site with comparison between sites using the chi-square test. Adherence to the PREHAB and PFMX prescriptions will be compared across study sites using independent samples t-tests. Estimates of efficacy (Group and Time main effect, as well as Group x Time interactions) will be analyzed using a repeated-measure analysis of covariance (ANCOVA), controlling for the baseline value of the outcome of interest and study site. Frequency of perioperative complications will be compared between the PREHAB and UC groups using chi-square analysis and as per the Clavien-Dindo classification system [58]. Length of stay between the PREHAB and UC groups will be compared using independent samples t-test.

## Discussion

The primary outcome of this study is to determine the feasibility of conducting a full-scale randomized controlled trial of PREHAB versus UC and to determine estimates of effect size to inform sample size for subsequent trials in this field. To our knowledge, this is the first study to examine a structured pre-surgical exercise program for men undergoing RP for PCa. This trial will advance our understanding of strategies to efficiently and effectively use the preoperative period to optimize postoperative recovery.

## Authors’ information

DSM is the Program Head of Kinesiology at the University of Guelph-Humber and Post-Doctoral Fellow at the Prostate Centre at the Princess Margaret Cancer Centre. AGM is a Senior Staff Psychologist and Clinician-Investigator in the Department of Surgery and the Department of Psychosocial Oncology and Palliative Care at the Princess Margaret Cancer Centre. WH is an MSc candidate at the University of Guelph, and a Kinesiologist and Research Assistant at the Prostate Centre at the Princess Margaret Cancer Centre. DA is an MSc candidate at the University of Guelph, and a Kinesiologist and Research Assistant at the Prostate Centre at the Princess Margaret Cancer Centre. RA is a Research Assistant with the Perioperative Program at the McGill University Health Centre. KE is MSc candidate at McGill University and a Research Assistant with the Perioperative Program at the McGill University Health Centre. SMHA is a Staff Physician in the Division of Internal Medicine and Geriatrics at the University Health Network and a Senior Scientist with Toronto General Research Institute. HC is a Staff Anesthesiologist and Medical Director of the Pain Research Unit at the Toronto General Hospital. PR is an Associate Professor in the School of Kinesiology and Health Science and Department of Psychology at York University and a Senior Scientist with Cancer Care Ontario. JT is a Professor of Surgery and Medical Imaging at the University of Toronto, a Staff Surgeon and Clinician Scientist in the Division of Urology and Director of the Prostate Centre at the Princess Margaret Cancer Centre. NEF is a Staff Surgeon, Clinician Scientist, and Head of the Division of Urology at the University Health Network and Princess Margaret Cancer Centre, and a Professor in the Department of Surgery at the University of Toronto. AF is a Staff Surgeon and Clinician Scientist in the Division of Urology at the University Health Network, and an Assistant Professor in the Department of Surgical Oncology with the University of Toronto. DW is a Staff Physician in the Department of Anesthesia and Pain Management with Toronto General Hospital and the University Health Network, Assistant Professor in the Department of Anesthesia and Institute of Health Policy Management and Evaluation with the University of Toronto, and a Clinical-Investigator in the Keenan Research Centre of the Li Ka Shing Knowledge Institute of St. Michael’s Hospital. AA is a Professor in the Department of Surgery and Urology at McGill University, Head of the Division of Urology and Department of Surgery at McGill University, Director of Cancer Care Mission, Head of the Department of Oncology at the McGill University Health Centre, and Urologist-in-Chief with the McGill University Health Centre. ST is a Professor in the Department of Surgery in the Division of Urology at McGill University, and is Director of Urologic Oncology at McGill University. FC is a Professor of Anesthesia and Associate Professor in the School of Dietetics and Human Nutrition at McGill University, and a Senior Staff Anesthesiologist at the McGill University Health Centre.

## Abbreviations

ANCOVA: Analysis of covariance
BF%: Body fat percentage
BMI: Body mass index
CHAMPS: Community Health Activities Model Program for Seniors
RP: Radical prostatectomy
FACT-F: Functional Assessment of Cancer Therapy-Fatigue
FACT-P: Functional Assessment of Cancer Therapy-Prostate
HADS: Hospital Anxiety and Depression Scale
HRQOL: Health-related quality of life
IIEF: International Index of Erectile Function
IPSS: International Prostate Symptom Score
PCa: Prostate cancer
PDI: Pain Disability Index
PFMX: Pelvic floor muscle exercise
PORPUS: Patient Oriented Prostate Utility Scale
PREHAB: Prehabilitation program
RC: Research coordinator
UC: Usual care
WC: Waist circumference
6MWT: 6-Minute Walk Test.
